# The mannose receptor (CD206) identifies a population of colonic macrophages in health and inflammatory bowel disease

**DOI:** 10.1038/s41598-021-98611-7

**Published:** 2021-10-04

**Authors:** Pamela B. Wright, Elizabeth McDonald, Alberto Bravo-Blas, Hannah M. Baer, Anna Heawood, Calum C. Bain, Allan M. Mowat, Slater L. Clay, Elaine V. Robertson, Fraser Morton, Jagtar Singh Nijjar, Umer Z. Ijaz, Simon W. F. Milling, Daniel R. Gaya

**Affiliations:** 1 Institute of Infection, Immunity and Inflammation, University of Glasgow, 120 University Place, Glasgow, G12 8TA UK; 2 MRC Centre for Inflammation Research, University of Edinburgh, Edinburgh, UK; 3 Gastroenterology Unit, Glasgow Royal Infirmary, Glasgow, UK

**Keywords:** Immunology, Inflammation, Innate immune cells, Mucosal immunology

## Abstract

To understand the contribution of mononuclear phagocytes (MNP), which include monocyte-derived intestinal macrophages, to the pathogenesis of inflammatory bowel disease (IBD), it is necessary to identify functionally-different MNP populations. We aimed to characterise intestinal macrophage populations in patients with IBD. We developed 12-parameter flow cytometry protocols to identify and human intestinal MNPs. We used these protocols to purify and characterize colonic macrophages from colonic tissue from patients with Crohn’s disease (CD), ulcerative colitis (UC), or non-inflamed controls, in a cross-sectional study. We identify macrophage populations (CD45^+^CD64^+^ HLA-DR^+^) and describe two distinct subsets, differentiated by their expression of the mannose receptor, CD206. CD206^+^ macrophages expressed markers consistent with a mature phenotype: high levels of CD68 and CD163, higher transcription of IL-10 and lower expression of TREM1. CD206^−^ macrophages appear to be less mature, with features more similar to their monocytic precursors. We identified and purified macrophage populations from human colon. These appear to be derived from a monocytic precursor with high CCR2 and low CD206 expression. As these cells mature, they acquire expression of IL-10, CD206, CD63, and CD168. Targeting the newly recruited monocyte-derived cells may represent a fruitful avenue to ameliorate chronic inflammation in IBD.

## Introduction

The incidence of inflammatory bowel disease (IBD) is increasing^1^ and around 1 in 200 adults are now affected. The two major clinically defined forms of IBD, Crohn’s disease (CD) and ulcerative colitis (UC), cause lethargy, abdominal pain, weight loss, rectal bleeding and diarrhoea^2^. IBD arises from a dysregulated immune response to the commensal gut microbiota in genetically susceptible individuals^3^.

To maintain health, the immune system in the gastrointestinal (GI) tract must maintain homeostasis by protecting the host from invasion of harmful pathogens, whilst simultaneously preventing potentially damaging responses against the commensal microbiota. Cells of the mononuclear phagocyte (MNP) lineage: monocytes, monocyte-derived macrophages, macrophages, and dendritic cells (DCs), are critical for this process. For example, MNPs expressing CD14 from the healthy intestine are refractory to cytokine stimulation whilst retaining phagocytic and bactericidal activity^4^. They are thus able to destroy invading bacteria without triggering damaging inflammation. However, aberrant MNP functions have been reported to contribute to IBD, including alterations in cytokine secretion, polymorphisms in molecules that sense bacteria (NOD2, Toll-like receptors (TLRs)), and dysregulation of transcription factors (e.g. NFκB)^5–7^.

Despite their importance, differential identification of the various MNP populations has been challenging because of the significant overlaps in their surface marker expression, and has generated much controversy^8^. Animal studies have provided the opportunity to examine the ontogeny of MNP populations. These have revealed that in most tissues, macrophages are self-sustaining embryonically-seeded populations, while DCs arise throughout life from a circulating precursor population. Cells phenotypically similar to macrophages and DCs can be generated from monocytes recruited into tissue^9^. Intestinal monocytes have the unusual capacity to generate all the sub-types of macrophage-phenotype cells. These include immature cytokine-producing populations that increase in frequency in mouse models of intestinal inflammation, and the mature unresponsive homeostatic populations^10,11^. Intestinal macrophages may therefore be more accurately described as monocyte-derived macrophages.

Much progress has already been made in identifying DC populations in the human intestine^12–17^, therefore, we focused on intestinal macrophage populations. Recent detailed descriptions of murine MNP populations have enabled clearer identification of these cells in samples from human tissue, including skin^18^, liver, lung, blood^19^ and lymph nodes^20^. Based on these findings the mannose receptor CD206 protein has been identified on macrophage populations in mice and humans, For example, in synovial tissue samples from patients with rheumatoid arthritis (RA), CD206^pos^ and CD206^neg^ macrophages appear to play different roles in driving inflammation or promoting tissue repair^21^, and are considered to be important therapeutic targets^22^.

Here we not only describe our strategy for the identification of human intestinal MNPs, but also provide novel data differentiating between intestinal macrophage populations based on their mannose receptor CD206 expression. We consider the functions of CD206^−^ and CD206^+^ colonic macrophages in healthy controls and patients with the inflammatory condition IBD, and identify CD206 as a marker to differentiate recently recruited cells from mature macrophages. Finally, we describe how, regardless of inflammatory status, both intestinal macrophage populations express Ki-67, a marker of recent proliferation. Despite this, they are not actively undergoing cell cycle. Thus, intestinal macrophages derive from a recently-proliferative precursor. Targeting these newly recruited cells may be a viable strategy for controlling intestinal inflammation.

## Results

### Identification of intestinal macrophage populations

Macrophages play a fundamental role in the pathogenesis of IBD. Advances in flow cytometry have permitted detailed immunophenotyping of leukocyte populations. We therefore designed multi-parameter flow cytometry panels to accurately identify myeloid cells residing within the intestinal lamina propria. Our initial aim was to develop a gating strategy to identify intestinal macrophages in healthy resected colonic tissues, which could be used to assess the phenotype and functions of these cells in subsequent experiments.

**Figure 1:**
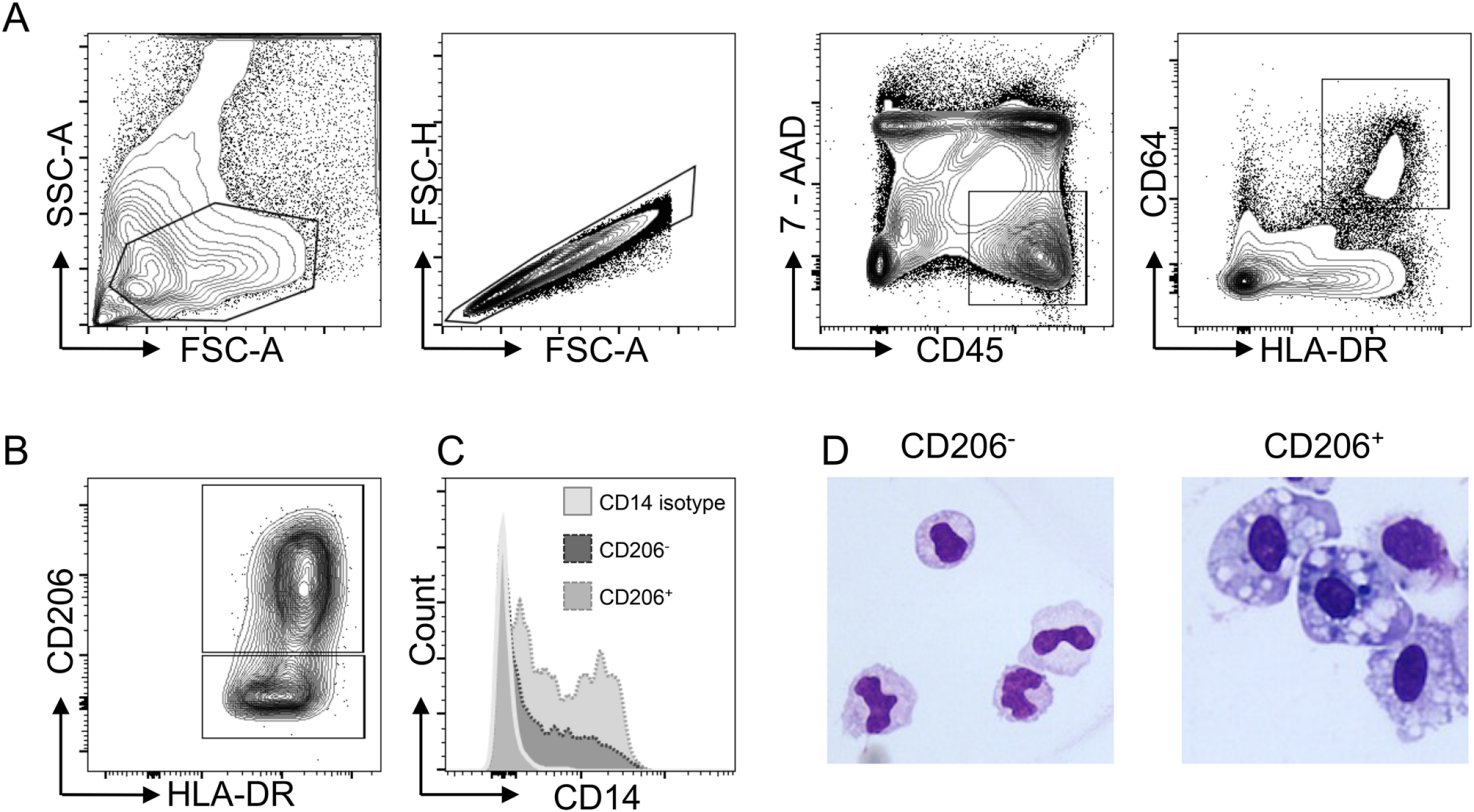
Identification of colonic human macrophage populations from healthy colon resections. (**A**) Gating strategy to identify human colonic macrophage populations by flow cytometry. Cells belonging to the monocyte: macrophage lineage reside within the live (7AAD-) single CD45 + CD64 + HLA-DR + leukocyte population. (**B**) Differential CD206 expression within CD64 + HLA-DR + population. (**C**) CD14 expression on CD64 + CD206 + and CD64 + CD206^−^ myeloid populations as compared to isotype control (histogram counts normalised to mode). (**D**) Morphological assessment of FACS-purified CD64 + HLA-DR + CD206^−^ and CD64 + HLA-DR + CD206 + cells. Data are representative of results from 10 samples.

Recent publications have shown conservation of surface marker expression between human and mouse, for example CD64, an Fc receptor, is a marker unique to both murine and human macrophages^13,23^. Thus, we examined CD45^+^ leukocytes for CD64 and HLA-DR expression (Fig. 1A). When characterising murine colonic MNP populations, previous reports have shown an upregulation of anti-inflammatory and downregulation of pro-inflammatory gene expression during the monocyte-to-macrophage differentiation process^10,13^. Specifically, a step-wise increase in the mannose receptor, CD206 can be seen in murine macrophages during their maturation, which we hypothesised would also occur in human intestinal macrophages. All CD64^+^ cells expressed HLA-DR (Fig. 1A), and thus CD64^+^ HLA-DR^+^ cells are defined as macrophages. As anticipated, CD64^+^ HLA-DR^+^ macrophages were heterogeneous for CD206 expression (Fig. 1B).

CD14 has been described as a marker for functionally-different macrophage populations^5^. Both CD64^+^ macrophage populations (CD64^+^ CD206^+^ and CD64^+^ CD206^−^) showed heterogeneous expression of CD14 (Fig. 1C). The majority of both CD206 + and CD206^−^ macrophages expressed CD14, but it was not differentially expressed between the two CD206 populations.

To further characterise these CD64^+^ CD206^+^ and CD64^+^ CD206^−^ macrophage populations, cells were flow cytometrically purified from healthy lamina propria tissue. Cytoplasmic vesicles, a defining feature of mature macrophages^24^, are clearly present within the cytoplasm of the CD206^+^ population, whereas the CD206^−^ cells displayed morphology consistent with a more immature macrophage phenotype (Fig. 1D).

Thus, we identified MNP populations within healthy intestinal lamina propria with a monocyte/macrophage phenotype. CD64^+^ CD206^+^ cells are the larger macrophage population; these contain cytoplasmic vesicles and exhibit heterogeneous CD14 expression. CD14 expression, more commonly associated with monocytes, has only recently been reported in small intestinal macrophages^15^. Our data are consistent with this finding, as we have identified CD14 expression on the majority of these cells.

### CD64+ CD206+ cells have the characteristics of mature macrophages

**Figure 2:**
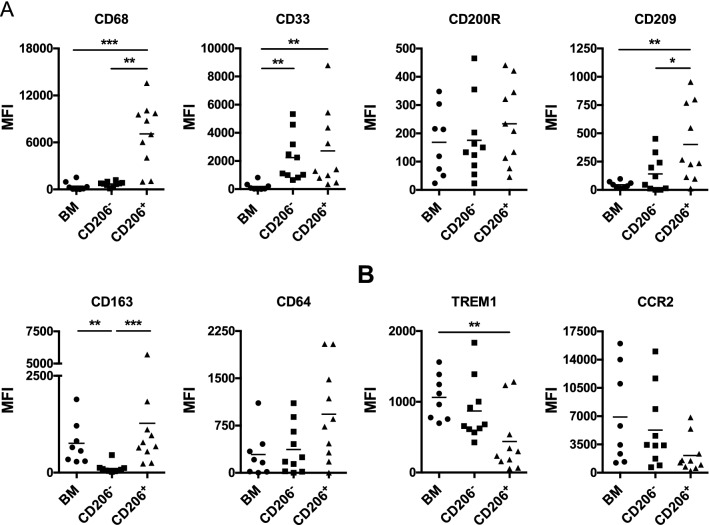
Phenotypic characterisation of CD64 + CD206^−^ and CD64 + CD206 + healthy colonic macrophages, and healthy blood monocytes (BM) by flow cytometry. (**A**) Expression of macrophage associated cell surface markers. (**B**) Protein expression of monocyte associated cell surface markers; n = 8, 10 and 10 for BM, CD206^−^ and CD206 + , respectively.

Several markers associated with the macrophage (Fig. 2A) and monocyte (Fig. 2B) lineages were investigated in CD64^+^ CD206^+^ and CD64^+^ CD206^−^ macrophages isolated from healthy colonic tissue resections. Classical blood monocytes (CD14^+^ CD16^−^) from healthy donors were used as a comparator population (Fig. 2). CD68 and CD33 have commonly been used as markers to identify macrophages^25^. CD64^+^ CD206^+^ macrophages expressed high levels of CD68, also a marker of cytoplasmic vesicles (Fig. 2A), supporting the morphological observations in Fig. 1D, both CD64^+^ CD206^+^ and CD64^+^ CD206^−^ macrophages express similarly higher levels of CD33 than blood monocytes. This indicates global expression of CD33 on human intestinal macrophages. In mice, expression of the inhibitory receptor CD200R is exclusive to mature intestinal macrophages^26^. However, our data show that in humans, CD200R expression does not differ between monocytes, and CD206^−^ or CD206^+^ macrophages. This suggests that it may be expressed in humans at all stages of intestinal macrophage differentiation (Fig. 2A).

A primary function of macrophages is to scavenge and phagocytose material from their external environment. Expression of the C-type lectin receptor CD209 is elevated in CD64^+^ CD206^+^ macrophages compared to their CD64^+^ CD206^−^ counterpart, as previously reported in mature macrophage populations of the human ileum^13^. While the scavenger receptor CD163 is known to be present on blood monocytes^27^, it is expressed on CD64^+^ CD206^+^ macrophages at higher levels than their CD64^+^ CD206^−^ counterparts (Fig. 2A). Thus, supporting our previous findings, CD206^+^ intestinal macrophages appear to express higher levels of molecules that enable mature macrophages to perform their phagocytic functions.

To better understand the relationship between blood monocytes and the CD64^+^ CD206^+^ and CD64^+^ CD206^−^ macrophage populations, we examined the levels of two markers highly expressed on blood monocytes (Fig. 2B), TREM1 and CCR2, with flow cytometry. Expression of TREM1 was found to be significantly lower on the CD64^+^ CD206^+^ macrophages than on blood monocytes, while the CD64^+^ CD206^−^ macrophage population appeared to express an intermediate level of TREM1 (Fig. 2B). There was also a trend towards lower expression of the chemokine receptor CCR2 in CD64^+^ CD206^+^ macrophages and CD64^+^ CD206^−^ macrophages, compared to blood monocytes, although this trend did not attain statistical significance. Therefore, as we expected, intestinal macrophages appear to lose expression of monocytic markers as they mature in the intestinal tissue.

These data indicate that CD64^+^ CD206^+^ cells are likely to represent mature human intestinal macrophages while the CD64^+^ CD206^−^ phenotype is consistent with an earlier differentiation state from blood monocytes that have recently entered the intestine. Work by Bernardo et al.^14^. proposed a similar maturation process in the development of intestinal macrophages from monocytic precursors. Bernardo reports initial expression and then gradual loss of CD11c as a marker of monocyte-macrophage differentiation. When we gated CD45^+^ CD64^+^ CD14^+^ macrophages, as described by Bernardo, we also observed cells with differential CD11c expression (Supplementary Fig. 1). Further analysis of the CD11c and CD206 expression in this macrophage subpopulation revealed a CD11c^hi^ sub-population expressing low levels of CD206. Gating on CD45^+^ CD64^+^ MHC II^+^ cells, using our usual strategy, also enables identification of this CD11c^hi^ CD206^−^ population of intestinal macrophages. These data are consistent with the idea that CD206^−^ cells are an earlier stage of differentiation than their CD206^+^ counterparts. The CD206^+^ cells can then be subdivided on the basis of their CD11c expression, as previously described^14^.

### *IL-10* expression by colonic macrophages correlates with CD206 expression

**Figure 3:**
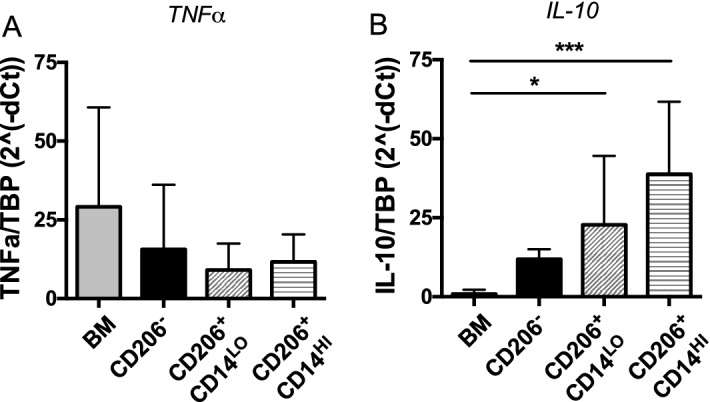
Expression of TNFα and IL-10 of FACS-purified healthy intestinal myeloid populations. (**A**) TNFα and (**B**) IL-10 expression were determined by qRT-PCR. CD64 + HLA-DR + CD206 + cells were subdivided into CD14LO and CD14HI populations. Expression of both cytokines was compared to blood monocytes (BM). Intestinal macrophages derived from normal colon resections; n = 6, 4, 8 and 10 for BM, CD206^−^, CD206 + CD14LO and CD206 + CD14HI, respectively. Error bars show + SD.

To understand how the functions of CD64^+^ CD206^+^ and CD64^+^ CD206^−^ macrophages differ, we examined their expression of transcripts for key cytokines, IL-10 and TNFα. IL-10 production is a characteristic feature of mature colonic macrophages^28^. To investigate the expression of TNFα and IL-10, we purified colonic myeloid populations from healthy tissue resections and CD14^+^ CD16^−^ blood monocytes from healthy donors using flow cytometry and measured their expression using qRT-PCR (Fig. 3). As described above, CD64^+^ CD206^+^ macrophages could be separated into two clear populations, identifiable by their differential expression of CD14. CD14-expressing human intestinal macrophages have previously been reported to contribute to intestinal inflammation, through production of cytokines including TNFα^5^. To assess whether these populations may be functionally different, we separated the CD206^+^ population into CD14^LO^ and CD14^HI^ sub-populations for analysis of their IL-10 and TNFα expression levels.

No significant differences were observed in the levels of TNFα transcripts produced by any of the populations, which matches previous results from both murine and human studies^13,14^ (Fig. 3A). However, both subsets of CD64^+^ CD206^+^ macrophages (CD14^HI^ and CD14^LO^) expressed significantly more IL-10 mRNA than blood monocytes. Our finding that the CD64^+^ CD206^+^ macrophages express the highest levels of IL-10 transcripts, independent of their level of CD14 expression, further supports the idea that these CD64^+^ CD206^+^ may be the most mature colonic macrophage population.

### CD206+ and CD206− macrophages show few intrinsic differences on a transcriptomic level

Our data are consistent with the idea that the CD64^+^ CD206^+^ and CD64^+^ CD206^−^ macrophage populations are at different stages in their differentiation; with the CD206^+^ macrophages representing the more mature, phagocytic and anti-inflammatory population. We therefore aimed to identify additional differences between our two identified macrophage populations, performing RNAseq analysis to assess each population’s global gene expression patterns. Using FACS, CD64^+^ CD206^+^ and CD64^+^ CD206^−^ macrophages were purified from six healthy regions of resected colonic lamina propria samples. The purified cells were then analysed by RNAseq. Reads were aligned using HISAT2 and all values expressed as normalised counts. As expected, the CD64^+^ CD206^+^ and CD64^+^ CD206^−^ macrophages were very similar in their overall gene expression profiles, as shown by principal component analysis (PCA), in which the CD64^+^ CD206^+^ and CD64^+^ CD206^−^ overlap (Supplementary Fig. 2).

Our analysis revealed a high degree of overall similarity between the macrophage populations, we observed only 8 genes from our total of 31,941 identified transcripts that were significantly altered (adjusted P-value < 0.05) between CD64^+^ CD206^+^ and CD64^+^ CD206^−^ macrophage populations (shown as a heatmap in Supplementary Fig. 2), indicating that the cells undergo only a small number of gene expression changes upon maturing and gaining CD206 expression. Interestingly, the 8 significantly altered genes were not those considered when investigating surface protein expression. No significant differences were observed in the gene expression of myeloid lineage markers (CD33, CD64, CD68, CD163, CD200R) between the CD64^+^ CD206^+^ and CD64^+^ CD206^−^ macrophages (Supplementary Fig. 3). However, there was a trend (though not significant) towards a positive correlation of genes involved in phagocytosis, scavenging and vesicle formation (*cd209*, *cd163* and *ap2a2*^29^), macrophage differentiation (CSF1R, MERTK^30^) and inhibition of innate immune response (*axl*^31^, *bcl2*^32^) in the mature CD64^+^ CD206^+^ macrophages compared to CD64^+^ CD206^−^. Data for genes encoding CD209 and CD163 were consistent with the data from our flow cytometric analyses (Fig. 2).

The gene with the most significant difference between CD64^+^ CD206^+^ and CD64^+^ CD206^−^ macrophages was serine/threonine/tyrosine interacting like 1 (*styxl1*), a gene of largely uncharacterised function (HGNC: HUGO Gene Nomenclature Committee), followed closely by Versican (*vcan*), which is enriched in inflamed tissues containing leukocytic infiltrate, including IBD intestinal mucosa^33,34^. Myotubulin-related protein 11 (*mtmr11*) and MCF.2 cell line derived transforming sequence like (*mcf2l*) are both elevated in CD64^+^ CD206^−^ colonic macrophages and their polymorphisms are associated with colonic carcinoma and osteoarthritis. Additional significantly differently expressed genes were ras-related protein 3D (*rab3d*), a gene upregulated during myeloid differentiation^35^ and zinc finger protein 296 (*zfp296*). The contributions of these genes to macrophage functions may be worthy of investigation, as they have not yet been fully explored.

**Figure 4:**
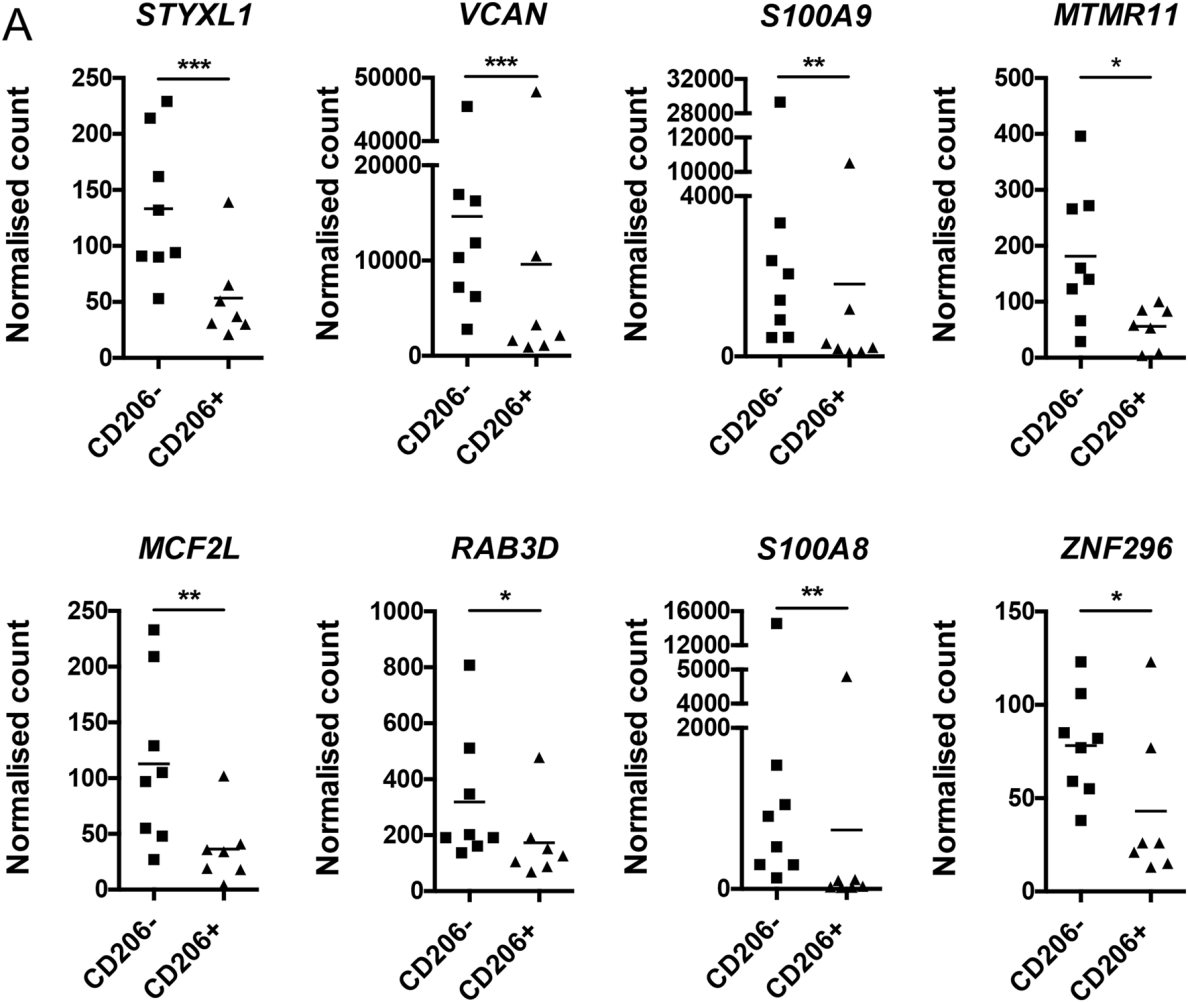
RNAseq analysis of FACS-purified CD206^−^ and CD206 + colonic human macrophage populations from healthy individuals. (**A**) Expression of differentially expressed genes between macrophage populations (CD206^−^ and CD206 +). Intestinal macrophages derived from normal colon resections; n = 8 and 7 for CD206^−^ and CD206 + , respectively.

Among the 8 genes with significantly altered expression (Fig. 4) are S100A8 and S100A9. These encode the proteins that together form the heterodimer calprotectin. This antimicrobial complex is known to be expressed by monocytes, macrophages, and neutrophils^36–38^. It can be released during inflammation and has become an established faecal biomarker in routine clinical IBD management. The higher expression of calprotectin genes in the CD64^+^ CD206^−^ macrophages is consistent with our hypothesis that these cells are the less mature, recently-recruited population that is yet to be conditioned into the homeostatic IL-10-producing mature macrophage population (Fig. 3B).

### Proportions of CD206^−^ and CD206^+^ macrophages in IBD

**Figure 5:**
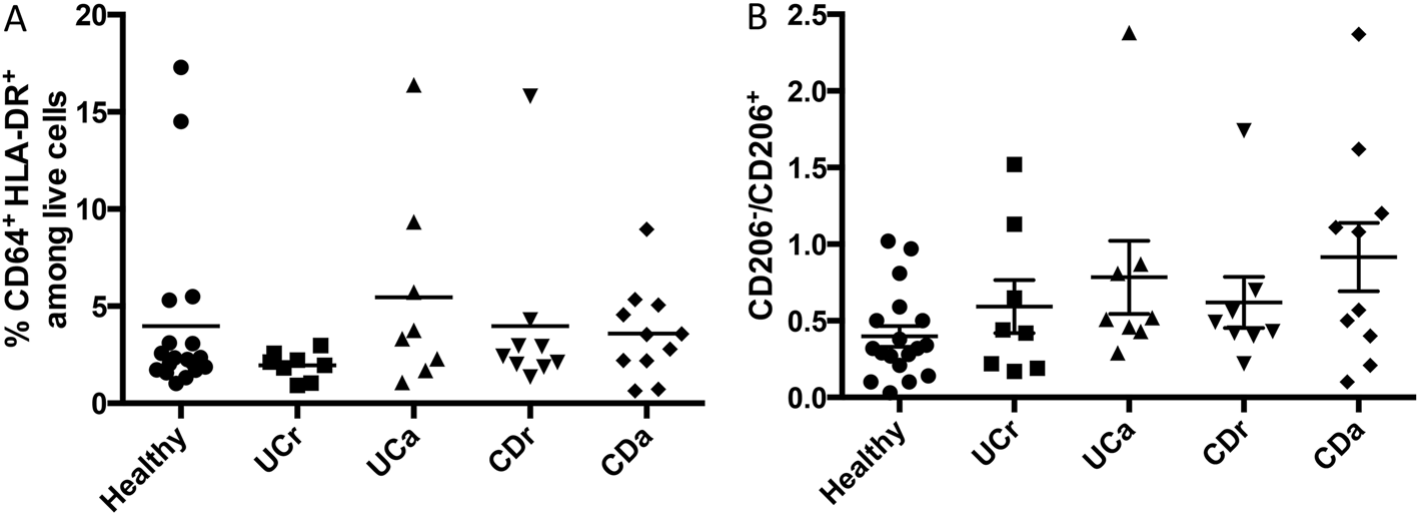
Intestinal macrophages in health and disease. (**A**) Proportion of total macrophages (CD64 + HLA-DR +) inthe lamina propria of healthy colonic tissue, and tissue isolated from CD and UC patients. (**B**) Ratio of CD206^−^:CD206 + macrophages. *UCr* UC tissue in remission, *UCa* active (inflamed) UC tissue, *CDn* CD tissue in remission and *CDd* active (inflamed) CD tissue. Sample numbers are n = 18, 8, 8, 9 and 10 for Healthy, UCr, UCa, CDr and CDa, respectively.

Mature macrophages play a critical role in the maintenance of intestinal immune homeostasis, while an increase in the proportion of the more immature cells is observed in models of intestinal inflammation^10,13^ and human studies^14,30^. Using our method of identifying human intestinal macrophages, we first assessed the proportion of total macrophages (CD45^+^ CD64^+^ HLA-DR^+^) among total live cells in colonic biopsies from both non-IBD controls (labelled as healthy) and from patients with CD and UC. Comparisons were made between macroscopically active (labelled as UCa and CDa) and in-remission (labelled as UCr and CDr) biopsies from IBD patients. Even though there are some samples with very high macrophage frequencies, particularly in the UCr and UCa groups, we do not observe significant differences in the overall macrophage frequency in any of the groups (Fig. 5A). Following this, CD64^+^ CD206^+^ and CD64^+^ CD206^−^ macrophages were enumerated in the same samples. The ratio of CD206^−^:CD206^+^ macrophages is shown in Fig. 5B. Statistically significant differences were not observed between the groups, either in the proportion of total macrophages, or in the ratio of CD206^−^:CD206^+^ macrophages (Fig. 5A,B). Unfortunately we were unable to purify sufficient macrophages from our biopsy samples to assess their production of inflammatory cytokines. Our data indicate that in most of our samples the CD64^+^ CD206^+^ population remains less frequent than their CD206^+^ counterparts, regardless of inflammatory status.

### Human colonic macrophages express Ki-67 but do not proliferate in situ

**Figure 6:**
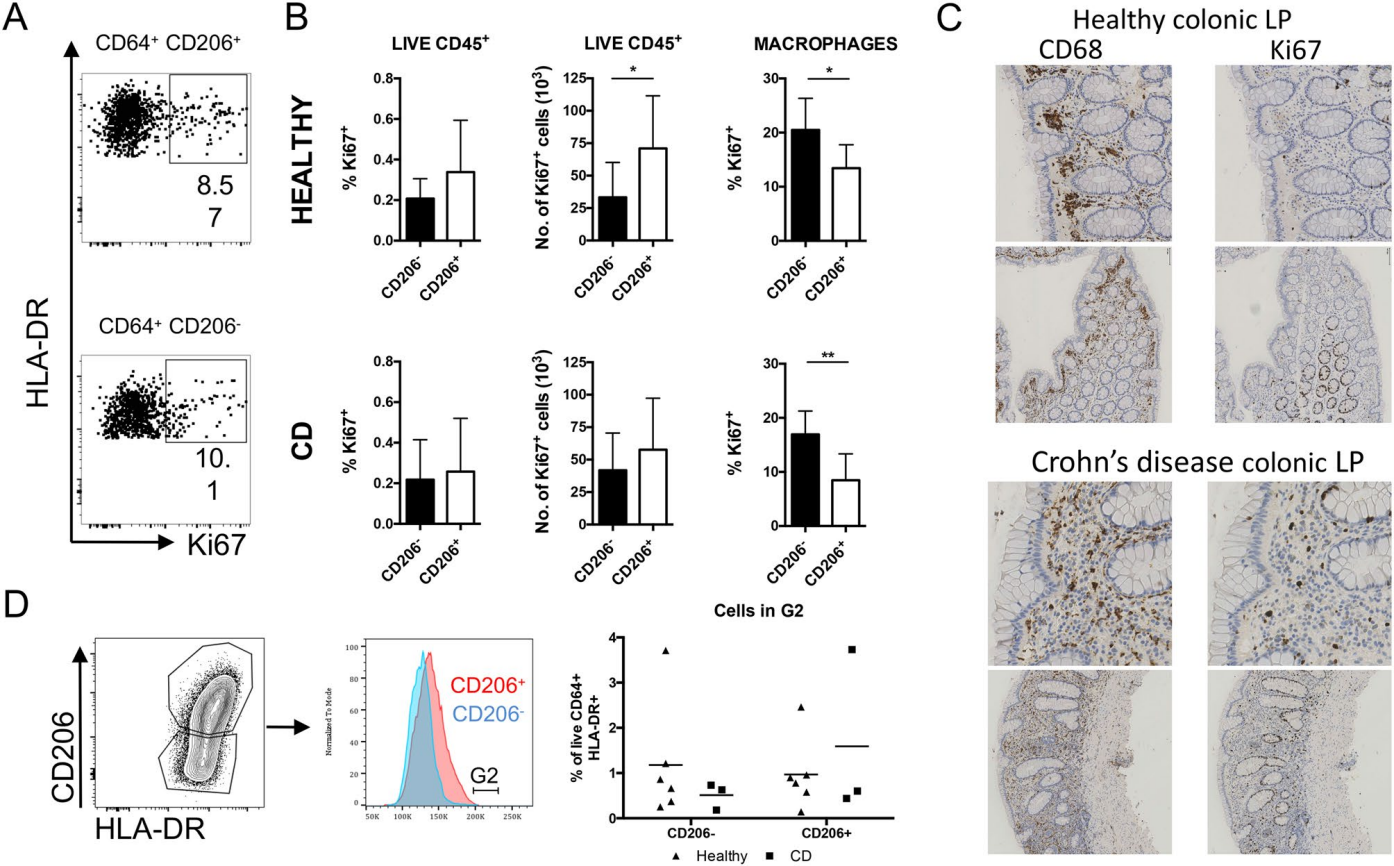
Proliferative capacity of human colonic macrophages. (**A**) Identification of human colonic Ki67 + macrophages isolated from lamina propria tissue of healthy colon biopsies. (**B**) Quantification of Ki67 + CD206 + and Ki67 + CD206^−^ macrophage populations in healthy and CD tissue. Error bars show + SD. n = 9 for both CD206^−^ and CD206 + . (**C**) Immunohistochemical staining of representative healthy and CD colonic cells cells for CD68 (left column) and Ki67 (right column). (**D**) Proliferation comparison between healthy and CD tissue. Example of gating, and frequencies of CD206^−^ and CD206 + macrophages in G2 in healthy and CD tissue.

Unlike most other tissue macrophages, the vast majority of adult intestinal lamina propria macrophages are generated from BM-derived monocytes. On reaching the intestine, murine monocytes differentiate through a series of intermediaries to give rise to mature macrophages that display very low levels of proliferation^39^. In addition, a small population of mature macrophages, identified by their TIM-4 expression, have been shown to be actively proliferative^40^. These data prompted us to look at the proliferative capacity of human colonic macrophages. Initially, Ki-67 expression was used to assess the proportion of colonic myeloid cells which have recently undergone proliferation (Fig. 6A–C and supplementary Fig. 4). Surprisingly, both CD64^+^ CD206^+^ and CD64^+^ CD206^−^ macrophage populations demonstrated significant expression of Ki-67 (Fig. 6A). 15–20% of colonic macrophages were Ki-67^+^ and Ki-67 expression was significantly higher in the less mature CD64^+^ CD206^−^ macrophages compared to the CD64^+^ CD206^+^ cells in both healthy colonic and inflamed CD colonic samples (Fig. 6B).

Histological staining of colonic tissue confirms results from our flow cytometric analysis (shown in Fig. 2A) showing that CD68^+^ cells are plentiful in colonic biopsies. CD68 is expressed by monocytes and macrophages, and if often used to identify macrophages in tissues^41^. In healthy tissue they are largely located adjacent to the epithelium, between the colonic crypts (Fig. 6C). In comparison, macrophages from active CD colonic biopsies can be observed among the infiltrating leukocytes in the inflamed tissue. When Ki-67 expression is examined, many proliferative cells are observed surrounding the colonic crypts, as expected^42^. In addition, numerous Ki-67^+^ cells can be seen in the locations occupied by macrophages, both in the healthy and CD biopsies, consistent with the results from our flow cytometric analysis in which we observe Ki-67^+^ macrophages in colonic biopsy tissue from both healthy and CD patients.

Because human colonic macrophages have previously been reported to be non-proliferative, and due to the possibility of Ki-67 expression persisting even when cells are arrested in G1/S or G2/M^43^, we performed cell cycle analysis by flow cytometry, quantifying DNA using DAPI. This analysis revealed that a negligible proportion of HLA-DR^+^ CD64^+^ macrophages were undergoing cell cycle (< 1% in G2), irrespective of CD206 expression (Fig. 6D). Thus, although human HLA-DR^+^ CD64^+^ colonic macrophages are not actively proliferating. However, their Ki-67 expression, especially in the immature CD206^−^ population, indicates that they may have recently differentiated from proliferative precursors.

## Discussion

MNP populations in the human intestinal mucosa are integral to the maintenance of tissue homeostasis and comprise one of the largest macrophage populations of the body. These intestinal macrophages are essential to the pathogenesis of IBD^44–46^. Characterising intestinal macrophage populations is therefore important for understanding how they shape inflammatory responses. Human intestinal macrophages have previously been identified by their expression of CD68 and CD33^5,47^. Multi-parameter flow cytometric analysis reveals conservation of many surface marker expression between mouse and human macrophages^48,49^. The Fc gamma receptor 1 (CD64) accurately identifies both human and murine gut macrophages expressing HLA-DR or MHCII^13,23^. We used this information to identify human intestinal macrophages (CD45^+^ CD64^+^ HLA-DR^+^) and find that CD206 can be used to distinguish between immature (CD206^−^) and mature (CD206^+^) macrophage populations; differences between these populations are consistent with their maturation in the intestine from a monocytic precursor population.

Murine studies have contributed greatly to the progress regarding phenotypic and functional characterisation of intestinal macrophages, mostly due to the availability of gene reporter animals, which facilitates specific cell identification. The human macrophage field has also recently made considerable progress^14–16,30^, however the complex phenotypic landscape of intestinal macrophages is still not fully understood. In this study, we distinguish two populations of CD64^+^ HLA-DR^+^ macrophages that can be differentiated based on their expression of CD206. CD206^+^ macrophages contain the vesicles that are characteristic of mature macrophages, and have significantly higher expression of CD163, CD68, and CD209, indicating that CD206 may be a useful maker to identify more mature colonic macrophage population in humans. Work by Bernardo et al.^14^, also described this intestinal monocyte to macrophage development process, reporting initial expression and then gradual loss of CD11c as a marker of monocyte-macrophage differentiation. Comparison of this approach with our own indicates that the CD206^−^ macrophage population all express high levels of CD11c, consistent with our interpretation that they are recently derived from monocytic precursors.

We were unable to purify sufficient cells from the available samples to perform functional assays. Therefore, to infer the functions of the CD206^+^ and CD206^−^ macrophage populations, we investigated their expression of transcripts encoding the cytokines IL-10 and TNFα. We find that a key anti-inflammatory cytokine, IL-10 had increased transcript expression in all macrophage populations compared to monocytes, with the highest gene expression in the CD206^+^ population. Macrophages are also capable of upregulating TNFα during inflammation^50,51^. Our data are consistent with reports that measured spontaneous TNFα production^14^ and show that both CD206^+^ and CD206^−^ macrophage populations are capable of expressing TNFα at similar levels.

Identification of these CD206^−^ and CD206^+^ macrophage populations enabled us to compare how they changed during intestinal inflammation. Surprisingly, our flow cytometric analyses did not reveal significant increases in the numbers of macrophages in inflamed tissue (Fig. 5A), or in the proportions of CD206^−^ and CD206^+^ macrophages in colonic biopsies from patients with IBD. Other investigators have reported increased proportions of less-mature monocyte-derived cells in inflamed samples from patients with IBD^17^. The difference between our data and other reports is unlikely to reflect differences in our patient population, as all samples designated as ‘active’ (CDa and UCa) were obtained from visibly-inflamed regions of the colon. Examination of tissue sections by immunohistochemistry revealed, as expected, that tissue architecture and localisation of macrophages within the tissue are dramatically altered by inflammation (Fig. 6D). We therefore speculate that lack of CD206 expression identifies a population of macrophages that have only recently entered the intestine, and that these recently-recruited CD206^−^ macrophages are able to partially mature and express CD206 even in regions of inflamed tissue.

In mice, intestinal macrophage populations are constantly replenished by blood monocytes and little proliferation of the recruited cells is observed, even in models of intestinal inflammation. In this study, close examination of human CD206^−^ and CD206^+^ macrophage subsets revealed that both populations contain a considerable proportion of Ki-67^+^ cells. This, however, may be a residual Ki-67 signal, as it was found in a higher proportion of CD206^−^ macrophages than in the mature CD206^+^ cells, in both healthy controls and in colonic biopsies from patients with CD. The fact that this Ki-67 expression did not indicate active proliferation was confirmed following flow cytometric analysis of macrophage DNA content, which showed that a negligible proportion of HLA-DR^+^ CD64^+^ cells were found to be in the S/G2 phases of the cell cycle.

Understanding the origins of intestinal macrophages and their differentiation may prove important for identifying the most effective therapeutic options in IBD. Our data show that, in humans, immature and more mature macrophage populations can be distinguished by their expression of CD206. Although we observe a macrophage-rich inflammatory infiltrate in inflamed colonic tissue, we were surprised to not observe consistent accumulation of the immature CD206^−^ cells in IBD patients. Our findings indicate that the earliest stages of the differentiation process, which include acquisition of CD206 expression, still occur efficiently even in inflamed colonic tissue affected by IBD. Thus, even when the normal intestinal monocyte-to-macrophage differentiation process fails to progress to the terminal tolerogenic state, the initial steps of this process still appear to occur. Therapeutic strategies designed to prevent migration of these CD206^−^ macrophages into inflamed tissues, or to influence the fate of this specific cell population, may therefore be effective in limiting macrophage dependent aspects of pathology in patients with IBD.

## Methods

### Patient cohorts and intestinal samples

Patients attending for colonoscopy at Glasgow Royal Infirmary were sent patient information sheets (requesting additional colonoscopic biopsies at the time of their forthcoming colonoscopy) along with their postal consent for the actual procedure itself. Signed informed consent for additional biopsy samples was taken on the day of the procedure by the principal investigator (DRG). All colonoscopies were undertaken following standard bowel preparation with KleanPrep, using Olympus 260 series colonoscope and the Olympus Lucera 290 series processor (Olympus, Tokyo). The biopsies were undertaken using Radial Jaw 4 reusable biopsy forceps (Boston Scientific, USA). Patients undergoing polyp surveillance colonoscopies were recruited as healthy controls and those with IBD, classed as either CD (active inflammation/diseased or in remission/normal) or UC (active inflammation/diseased or in remission/normal). Histologically normal margins of surgically resected intestinal material were also obtained as additional healthy controls; all healthy margins used were from colorectal cancer resections. Following collection, samples were stored on ice in sterile PBS during transport to the lab for immediate analysis. All methods were performed in accordance with the relevant guidelines and regulations. Samples were managed by the National Health Service Greater Glasgow and Clyde (NHSGGC) Research Tissue Bank, under ethics granted by the West of Scotland Research Ethics committee 4, (REC 14/WS/1035).

### Intestinal tissue processing

To remove the epithelial layer from biopsies, tissue was incubated in 2 mM EDTA/HBSS (Gibco) for three minutes at 37 °C in a shaking incubator. For resected tissue samples, fat and muscle layers were first removed; tissue was washed in HBSS and subsequently dissected into 0.5 cm pieces. To remove the epithelial layer from resections, tissue was incubated in 2 mM EDTA/HBSS for 15 min at 37 °C in a shaking incubator. Tissue was vigorously shaken; supernatant discarded and the process was repeated three times. After the final EDTA step, all samples were washed in HBSS and resuspended in 2 ml (20 ml for resections) of pre-warmed 10% complete media (RPMI 1640 supplemented with 10% foetal calf serum (FCS), 100 U ml^−1^ penicillin, 100 U ml^−1^ streptomycin, 2 mM L-glutamine and 50 μM 2-mercaptoethanol) containing: Collagenase VIII (1 mg/ml, Sigma-Aldrich), Collagenase D (1.25 mg/ml, Roche), Dispase (1 mg/ml, Gibco) and DNase. Samples were returned to the shaking incubator (37 °C) for 30–40 min; additional vigorous shaking was performed every 10 min to aid digestion. Cell suspension was first filtered through a 100 μM then a 40 μM cell strainer (BD Falcon) and washed in PBS containing 2 mM EDTA and 2% FCS.

### Flow cytometry

Following Fc receptor blocking (eBioscience), antibody staining was performed in PBS with 2% FCS and 2 mM EDTA. Where biotin-conjugated antibodies were used, fluorochrome-labelled streptavidin was used to visualise antibody binding. Samples were acquired using an LSR II (BD Biosciences) flow cytometer, or purified using a FACSAria II/III cell sorter (BD Biosciences). Data were analysed using FlowJo software (Tree Star, Ashland, OR). For Ki-67 staining, cells were fixed for 30 min at room temperature and then permeabilised (eBioscience intracellular staining kit). Cells were subsequently incubated with Fc block and intracellular cytokine staining was performed.

### Flow cytometry antibodies

Directly conjugated antibodies targeting CD45 (HI30), CD64 (10.1), HLA-DR (L243), CD206 (15–2), CCR2 (K036C2), CD33 (WM53), TREM1 (TREM-26), CD43 (10G7), CD200R (OX-108), CD64 (10.1), αVCD64 (10.1), CD68 (Y1/82A), CD14 (M5E2), CD16 (3G8), CD14 (M5E2), CD11c (Bu15), CD3 (UCHT1), CD15 (W6D3), CD19 (HIB19), CD56 (MEM-188), SIRPα (SE5A5), CD115 (CSF-1R), CD1c (L161), CD11b (ICRF44) and 7AAD were from Biolegend. CX3CR1 (2A9-1) from MBL International, CD163 (GH1/61), CD103 (B-Ly7) and CD209 (eB-h209) from ebioscience and Ki-67 (B56) from BD biosciences were also used.

### Immunohistochemistry

Following fixing in 10% formalin, intestinal biopsies were processed in a 2 h xylene cycle (room temperature for 2 min 70% ethanol, 2 min 90% ethanol, 2 min 100% ethanol; 45 °C for 11 min 90% ethanol and 30 min 100% ethanol; room temperature for 2 min in xylene; 45 °C for 28 min in xylene, 65 °C for 30 min in wax with vacuum) and stored at NHSGGC biorepository.

### Immunohistochemistry antibodies

Antibodies targeting Ki-67 (MIB-1 (1/400)) and CD68 (PG-M1 (1/200)) from DAKO and FXIIIa (E980.1) from LEICA were used for immunohistochemistry staining.

### RNA extraction and real-time quantitative PCR

The MicroRNA kit (Qiagen) was used to extract RNA. Genomic DNA was removed and cDNA was generated using the Superscript First Strand kit (Invitrogen).

Gene expression was measured using the Brilliant III Ultra Fast SYBR qRT-PCR Master Mix (Agilent Technologies). Reactions were analysed on the 7500 Fast Real-Time PCR System machine (Applied Biosystems). Primers used were TBP: forward AGACCTTCCTGTTTACCCTTG, reverse TAGCTGTGGGTGACTGCTTGG, IL-10: forward AAGACCCAGACATCAAGGCG, reverse AATCGATGACAGCGCCGTAG and TNFα: forward CACCACCATCAAGGACTCAA, reverse GAGGCAACCTGACCACTCTC. Expression levels were normalised to TBP. Results were presented as 2^-ΔExt)^.

### RNASeq analysis

#### Sample preparation

Histologically normal margins of healthy colonic resections were processed, digested and stained for FACS analysis as previously described. CD45^+^ CD64^+^ HLA-DR^+^ CD206^+^ and CD45^+^ CD64^+^ HLA-DR^+^ CD206^−^ cells were sorted using a FACSAria II/III. Cells were sorted into 300 μl RLT buffer (Qiagen) and stored at − 80 °C prior to RNA extraction using a MicroRNA kit (Qiagen).

#### Data acquisition

Samples were transported on dry ice to Glasgow Polyomics research facility at the University of Glasgow. The sequencing libraries were prepared using the SMARTer Stranded Total RNA-Seq kit-Pico Input Mammalian (Clontech), starting with 1 ng of RNA and following the manufactures protocol with 15 cycles of PCR. Subsequently, the sequencing was done on the Illumina NextSeq 500 using High Throughput flow-cells multiplexing eight samples per flow cell to produce 50 M read-pairs per sample on average. The sample-specific fastq files were generated using bcl2fastq software.

#### Statistical analysis

Data was aligned using HISAT2 and the reads summarised and normalised using the Feature Counts tool. Differential expression was performed using the DESeq2 R package. Significant differential expression was defined as adjusted P-value < 0.05. Original data are available from the Gene Expression Omnibus (www.ncbi.nlm.nih.gov/geo/ study GSE124350).

### Statistics

Results are presented as mean for grouped samples. Data were first checked for Gaussian distribution by Shapiro–Wilk normality test. Data were then analysed by Student’s t test (for normally distributed) or Mann Whitney (for non-parametric). Or, for multiple comparisons, a one-way ANOVA (for normally distributed) or Kruskal Wallis Spearman correlative tests (for non-parametric) were performed, with Dunn multiple comparisons post test. Values of p < 0.05 were considered statistically significant. Statistical analysis was performed using GraphPad Prism.

## Supplementary Information

Supplementary Information.

## Abbreviations

CD: Crohn’s disease
DC: Dendritic cell
IBD: Inflammatory bowel disease
MNP: Mononuclear Phagocyte
UC: Ulcerative colitis
