# Supplementary material for: The mannose receptor (CD206) identifies a population of colonic macrophages in health and inflammatory bowel disease
Source: Sci Rep. 2021 Oct 4;11:19616. doi: 10.1038/s41598-021-98611-7 (PMC8490356; doi:10.1038/s41598-021-98611-7)
Supplement: Supplementary file 1 — Supplementary Information. [file 41598_2021_98611_MOESM1_ESM.pdf]

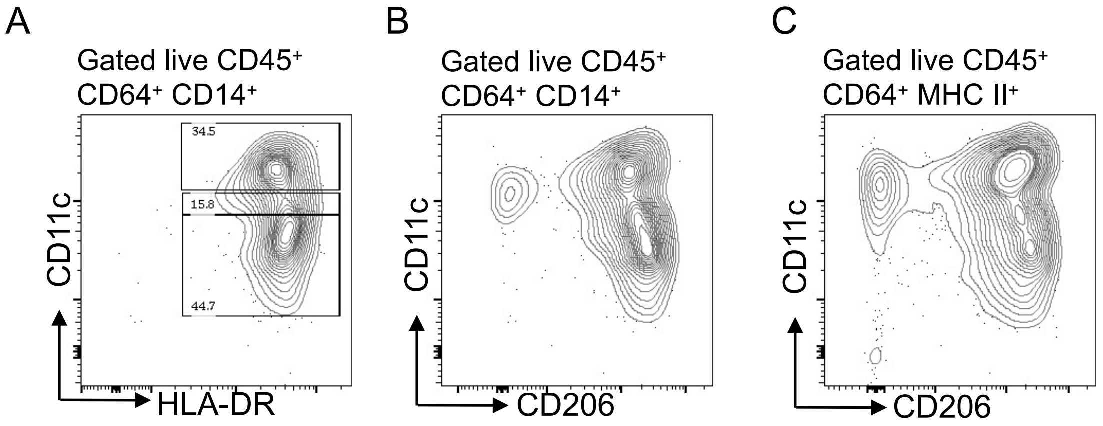

Supplementary Figure 1. Staining of CD45+ CD64+ CD14+ cells and CD45+ CD64+ MHC II+ cells to investigate the relationship between CD11c and CD206 staining.

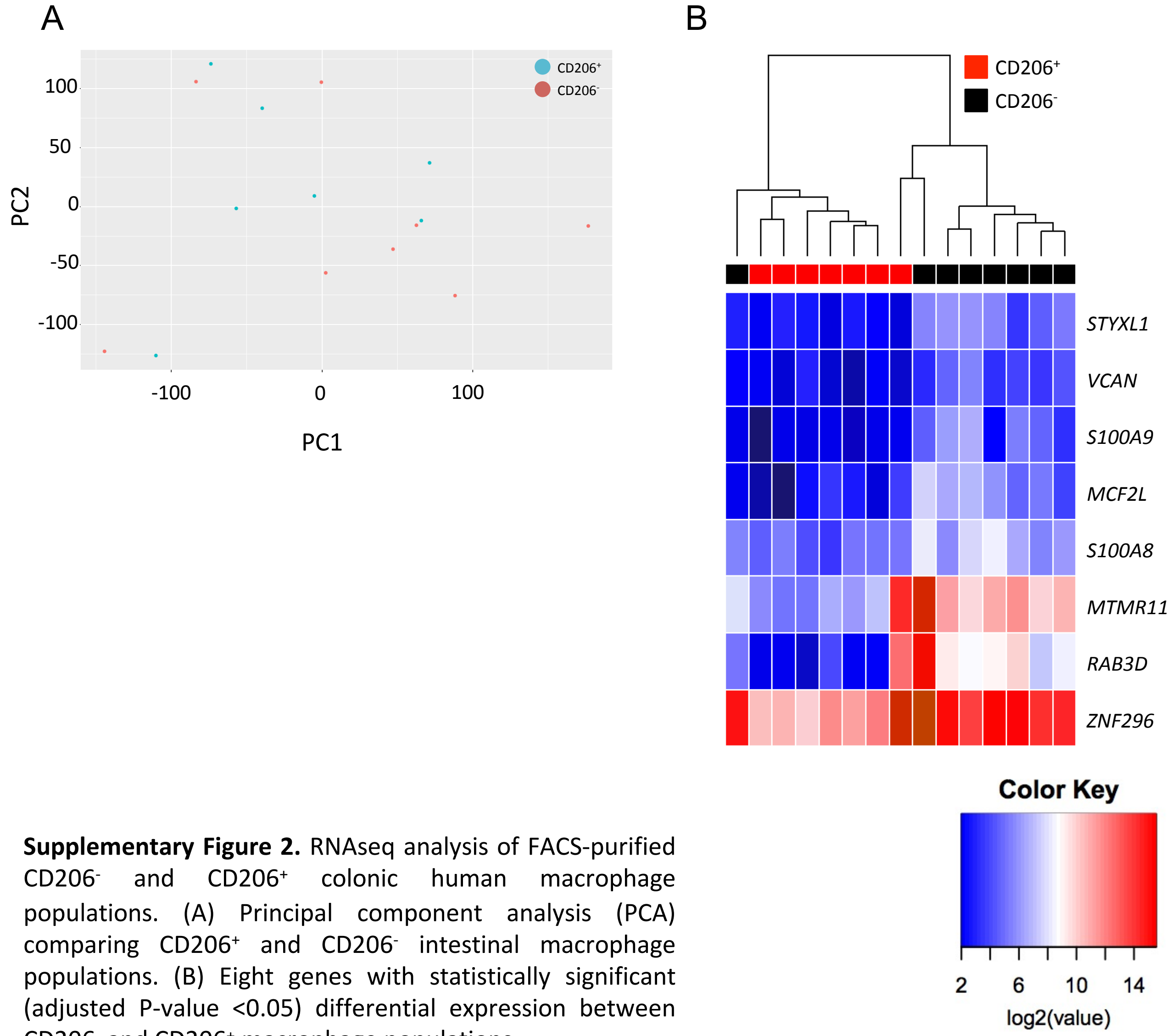

Supplementary Figure 2. RNAseq analysis of FACS-purified CD206^{-} and CD206^{+} colonic human macrophage populations. (A) Principal component analysis (PCA) comparing CD206^{+} and CD206^{-} intestinal macrophage populations. (B) Eight genes with statistically significant (adjusted P-value <0.05) differential expression between CD206^{-} and CD206^{+} macrophage populations.

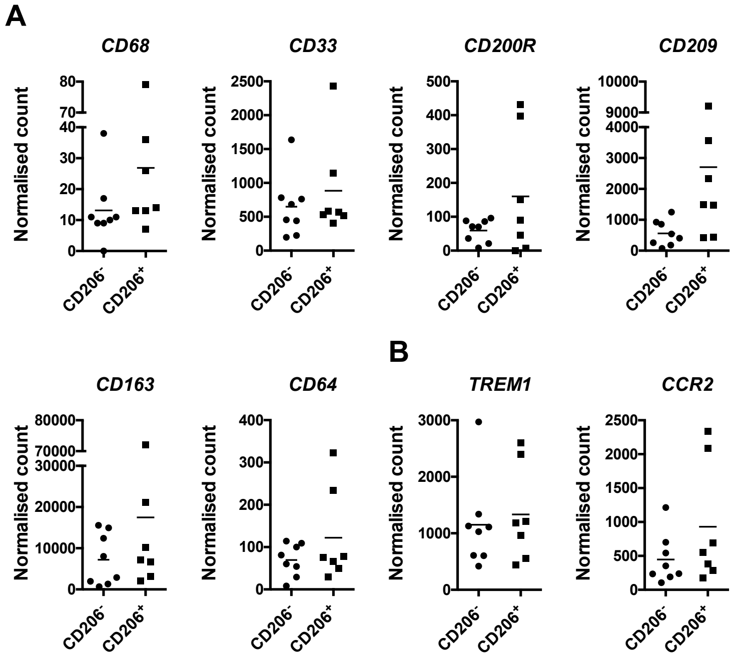

Supplementary Figure 3. RNAseq analysis of FACS-purified CD206- and CD206+ colonic human macrophage populations. (A) Comparative expression of macrophage associated cell surface markers on CD206- and CD206+ cells. (B) Comparative expression of monocyte-associated cell surface markers on CD206- and CD206+ cells. Intestinal macrophages derived from healthy colon resections; n=8 and 7 for CD206- and CD206+, respectively.

A

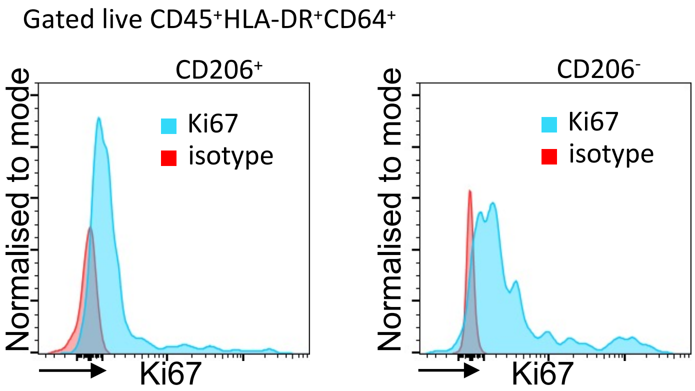

Supplementary Figure 4. Ki67 staining from CD206+ and CD206- compared to their isotype control (red).
